# The influence of the number of screws and additional surgical procedures on outcome in hallux valgus treatment

**DOI:** 10.1186/s13018-018-0796-z

**Published:** 2018-04-25

**Authors:** Thorsten Jentzsch, Niklas Renner, Richard Niehaus, Jan Farei-Campagna, Marcel Deggeller, Fabrice Scheurer, Katie Palmer, Stephan H. Wirth

**Affiliations:** 1 Department of Orthopaedics, Balgrist University Hospital, University of Zurich, Forchstrasse 340, 8008 Zurich, Switzerland; 2 University of Zurich, Zurich, Switzerland; 3 San Camillo Hospital IRCCS, Venice, Italy

**Keywords:** Hallux valgus (HV), Reversed L-shaped osteotomy (ReveL), Long plantar arm osteotomy, Screws, Recurrence, Patient satisfaction

## Abstract

**Background:**

Surgical treatment of hallux valgus (HV) is one of the major flagships of orthopedic surgeons. Due to relatively unsatisfactory radiological and clinical outcomes, the search for the best surgical technique and causes for unsatisfactory outcomes continues. The objective was to investigate associations of the number of screws and additional surgical techniques for HV with radiological and clinical outcome after reversed L-shaped osteotomy (ReveL).

**Methods:**

A retrospective cohort study of adults from a single University Hospital between 2004 and 2013 was performed. The primary exposure was the number of screws (one vs two) used for osseous fixation after ReveL. The secondary exposure was an additional surgical technique for HV (e.g., Akin osteotomy). The primary outcome was a radiological recurrence of HV (HV angle (HVA) > 15°) at last follow-up. The secondary outcomes were limited patient satisfaction, complication, revision surgery, and elective hardware removal. Odds ratio (OR) and 95% confidence interval (CI) were estimated by logistic regression adjusting for confounders.

**Results:**

The recurrence was 45% less likely with the use of one screw, independent of age, sex, additional technique, and preoperative HVA (odds ratio (OR_adjusted_) = 0.55 [95% CI 0.30–0.98], *p* = 0.043). The recurrence was 162% more likely with an additional surgical technique for HV (OR_adjusted_ = 2.62 [1.24–5.52], *p* = 0.011).

**Conclusion:**

In ReveL for HV, a single screw (instead of two screws) may be sufficient enough for a similar or even better outcome, which may also reduce costs. Additional surgical procedures for HV may be refrained from if possible. Due to limitations of a retrospective study, results may need validation with clinical trials.

## Background

Hallux valgus deformity (HV) is a very common disease, affecting about every fourth individual up to 65 years and around every third individual later on in life [1]. Patient satisfaction after HV surgery (77.4%) is lower than that in other typical orthopaedic procedures, such as total hip arthroplasty (91.9%) [2]. Thus, the search for the optimal surgical technique and causes for unsatisfactory outcomes remains of current interest.

There are many different surgical techniques for HV [3]. Instead of using a V-shaped osteotomy [4], the distal metatarsal (biplanar) reversed L-shaped osteotomy (ReveL) utilizes a short dorsal vertical and long plantar horizontal osteotomy providing high corrective power and intrinsic mechanical stability [5–7]. A tarsometatarsal arthrodesis with two crossed screws may be added in cases, where hypermobility of the first tarsometatarsal joint is observed during clinical examination [8–10]. A phalangeal Akin osteotomy with one screw can also be used as an additional procedure to obtain more angular and rotational correction [11, 12].

Although unknown for the distal metatarsal bone, in the proximal metatarsal bone, long plantar arm osteotomies may have less stability than short plantar arm osteotomies [13]. Therefore, in theory, strong fixation of a distal metatarsal osteotomy may be needed in order to avoid osteotomy failure and recurrence of HV. Although the use of one screw has been mainly reported in the literature, in theory, two screws offer a stronger fixation than one screw. In line with this assumption, two screws have been associated with faster time to healing of osteotomies after chevron osteotomy (*n* = 52) [14]. However, a recent study has not found any differences in time to healing of osteotomies after chevron osteotomy using one or two screws (*n* = 75) [15]. Two screws are not only more expensive but also bulkier, which may lead to tissue irritation and hardware removal. Furthermore, additional surgical techniques for HV may help correcting greater deformities [16]. However, it remains unknown if the number of screws and additional surgical techniques for HV are associated with radiological and clinical outcome after long plantar arm osteotomies.

Previous studies about long plantar arm osteotomies did not investigate the chosen exposures, i.e., the number of screws and additional surgical techniques for HV on unsatisfactory radiological and clinical outcomes. The objective of the current study is to investigate associations of the number of screws and additional surgical techniques for HV with radiological and clinical outcome after ReveL for HV.

## Methods

### Study design

A retrospective cohort study with a superiority design and an internal comparison group was performed. It was part of a Master’s thesis, and the results about different study aims are described elsewhere [17–19]. Patient charts with radiological and clinical data from the Balgrist University Hospital in Switzerland between January 2004 and December 2013 were examined. Ethics approval was given by the local ethics committee (cantonal ethics committee Zurich 2015-0480), allowing this retrospective study with a large number of patients to be performed without the need for individually signed informed consent.

### Surgical technique

The surgical technique is described in more detail elsewhere [18]. Briefly, surgery was only performed for painful HV. ReveL was carried out with a short dorsal vertical and long plantar horizontal osteotomy (beginning dorsally 1 centimeter (cm) proximal to the metatarsophalangeal (MTP) joint, then changing direction to a proximally directed osteotomy in the middle of the dorsoplantar distance before exiting the plantar cortex about 4 cm proximal to the MTP joint; keeping the biplanar option of medial wedge resection when needing decrease of the distal metatarsal angle). Fixation was achieved with one or two 2.4 mm cortex screws directed dorsoplantarly, and postoperative care consisted of unrestricted ambulation in a postoperative shoe with a rigid sole for 4–6 weeks [20].

### Exposure and outcome measures

**Fig. 1:**
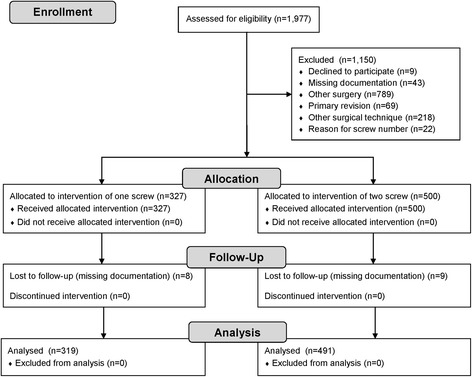
Flow chart of case enrolment in this retrospective cohort study between January 2004 and December 2013 [34]

The inclusion criteria were primary ReveL for HV in adult patients. The local database (KISIM; CISTEC AG, Zurich, Switzerland) was searched for the key term “hallux valgus” (Fig. 1). Several exclusion criteria were applied. Eventually, 827 cases were investigated in this study. Patient charts were investigated utilizing the local database (KISIM) and imaging software (IMPAX 6.4.0.6010 and IMPAX Orthopaedic Tools, Agfa-Gevaert N.V. (Agfa), Mortsel, Belgium). The data were gathered anonymously with REDCap (version 6.11.5; Vanderbilt University, Nashville, TN, USA) and cleaned with Microsoft Excel (version 2010; Microsoft Corporation, Redmond, WA, USA) as well as Stata/IC (version 13.1; StataCorp LP, College Station, TX, USA).

**Fig. 2:**
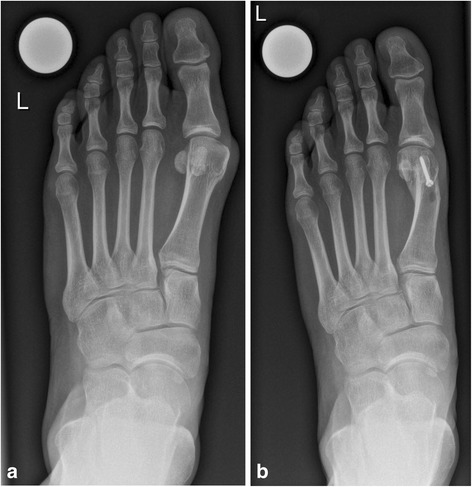
Conventional dorsoplantar radiographs of the left foot in a 37-year-old female. **a** Preoperative radiograph showing a hallux valgus deformity. **b** Postoperative radiograph showing correction of a hallux valgus deformity 1 year after reversed L-shaped osteotomy (ReveL) using one screw

**Fig. 3:**
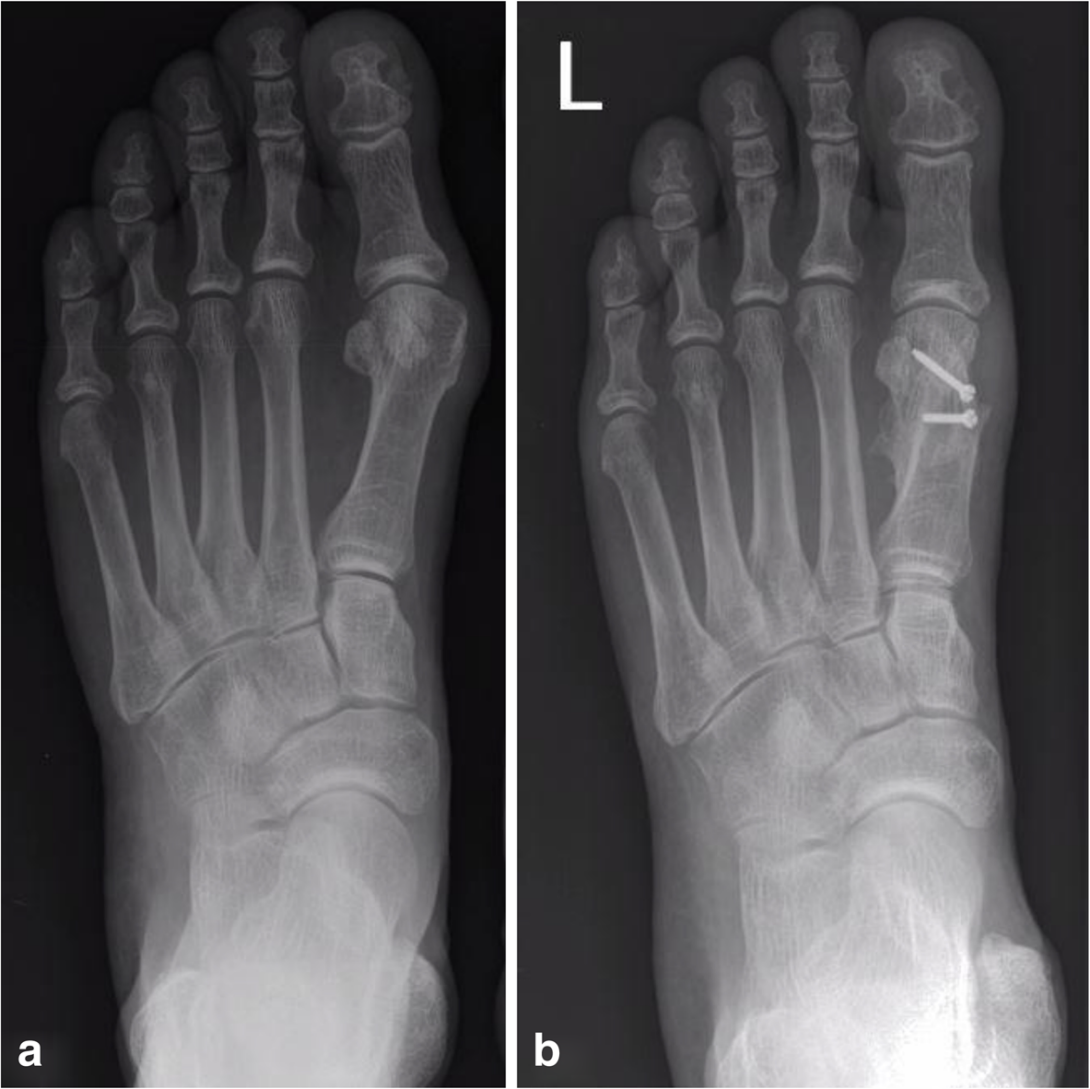
Conventional dorsoplantar radiographs of the left foot in a 60-year-old male. **a** Preoperative radiograph showing a hallux valgus deformity. **b** Postoperative radiograph showing correction of hallux valgus deformity 1 year after reversed L-shaped osteotomy (ReveL) using two screws

The main exposure variable was the number of screws (one or two) used for fixation of the osteotomy during ReveL (Figs. 2 and 3). The secondary exposure variable was the use of additional surgical techniques for HV (no or yes), which consisted of tarsometatarsal arthrodesis (no or yes) and/or Akin osteotomy (no or yes). A tarsometatarsal arthrodesis was added if clinical examination revealed hypermobility of the first ray in the tarsometatarsal joint. An Akin osteotomy was performed if HV interphalangeus (lateral deviation of the distal phalanx in the interphalangeal joint) was found. As explained in Tables 2 and 3, other documented exposure variables, potential confounders, and/or effect modifiers were considered. The time periods were chosen according to the local changes in staff that occurred in the foot and ankle team in 2007 and consecutive changes in personal preferences for the use of one or two screws. The performance of an additional surgical procedure on the foot consisted of any other simultaneous surgery on the foot, such as the Hohmann technique (proximal phalangeal head resection of the lesser toes) for hammer foot deformity or Coughlin osteotomy (longitudinal diaphyseal osteotomy of the fifth metatarsal bone) for Bunionette deformity [21, 22].

The primary (categorical) outcome variable was the radiological recurrence of HV (HV angle (HVA) > 15°) at last radiological follow-up [23]. The secondary (categorical) clinical outcome variables were limited patient satisfaction, complication, revision surgery, and elective hardware removal.

### Statistical methods

The data were non-normally distributed according to the skewness and kurtosis test for normality, and medians (interquartile range (IQR)) are provided. Categorical data were cross-tabulated and assessed with the chi-squared or Fisher’s exact test. Odds ratios (OR) and 95% confidence intervals (CI) were calculated for each category. Confounders were considered to be factors associated with the radiological recurrence of HV and the number of screws after ReveL without being on the causal pathway between both variables. A priori, age and sex were defined as confounders since they commonly influence the prevalence of diseases, including HV [24–27]. Preoperative HVA was also chosen as an a priori confounder since it represents the severity of HV and influences the recurrence of HV after surgery [28]. Further confounding was assumed when the crude and adjusted estimates differed by ≥ 10%. Effect modification was present when stratum-specific estimates differed according to the test for heterogeneity. Adjusted Mantel-Haenszel estimates for potential confounders and stratum-specific estimates for effect modifiers were calculated for the association between radiological recurrence of HV and the number of screws after ReveL. Likewise, secondary outcomes as well as the remaining exposure variables were assessed accordingly. To study the objectives of associations between the number of screws after ReveL and additional surgical techniques for HV with radiological recurrence of HV, logistic regression was used to obtain a causal model. Likelihood ratio tests were used to compare different models with and without interaction terms and linear trends. A Wald test assessed differences in the odds in different categories of a single variable.

Among others, bias was limited by calculation of a sample size (type II error), exclusion of cases with a reason for a specific number of screws (selection bias), hypothesis testing without knowledge of surgeons and patients (blinding, performance, and reporting bias), adjusting for confounders including time period as a surrogate for changes in staff and preferences for the number of screws (performance bias), and disclosure of missing values (selection bias). A clinically relevant difference in radiological recurrence of 10% would be important to detect. At a significance level of 5% and power of 80%, this required ≥ 199 cases per group. Statistical significance was assumed at a 5% level. Analyses were carried out with Stata/IC (StataCorp LP).

## Results

### Participants

At a median follow-up of 12.0 (IQR 4.0–19.0) months, 17 (2.1%) cases were lost to follow-up and 810 (97.9%) cases analysed. There were no differences between those that were lost to follow-up and those included in the study regarding the number of screws (*p* = 0.522).

While two screws were more commonly used in the earlier time period (one screw, *n* = 53 [11.5%] vs two screws, *n* = 409 [88.5%], *p* < 0.001), one screw was more commonly used in the second period (one screw, *n* = 266 [76.4%] vs two screws, *n* = 82 [23.6%], *p* < 0.001).

### Exposure variables

More cases were treated with two screws after ReveL (*n* = 491 [60.6%]) compared to one screw (*n* = 319 [39.4%]) (Table 1). The stand-alone use of ReveL for HV (*n* = 514 [63.5%]) was more common than additional surgical techniques for HV (*n* = 296 [36.5%]). An additional Akin osteotomy (*n* = 289 [35.7%]) was more common than tarsometatarsal arthrodesis (21 [2.6%]). The majority of cases did not receive additional surgery on the remaining foot (*n* = 496 [61.2%]).

**Table 1:** Exposure variables of cases treated with reversed L-shaped osteotomy (ReveL) for hallux valgus deformity (*n* = 810)

Variable	Category	*N*_total_ (%)	*N*_category_ (%)
One screw after ReveL		810 (100.0)	
	No		491 (60.6)
	Yes		319 (39.4)
Additional surgical technique for HV^*^		810 (100.0)	
	No		514 (63.5)
	Yes		296 (36.5)
Additional tarsometatarsal arthrodesis		810 (100.0)	
	No		789 (97.4)
	Yes		21 (2.6)
Additional Akin osteotomy		810 (100.0)	
	No		521 (64.3)
	Yes		289 (35.7)
Time period		810 (100.0)	
	2004–2007		462 (57.0)
	2008–2013		348 (43.0)
Additional surgery on foot^†^		810 (100.0)	
	No		496 (61.2)
	Yes		314 (38.8)

Abbreviations: *N* number, *%* percent, *IQR* interquartile range, *ReveL* reversed L-shaped osteotomy, *HV* hallux valgus deformity

^*^Tarsometatarsal arthrodesis or Akin osteotomy

^†^Any other surgery on the foot except for HV (e.g., Hohmann technique of lesser toes or Coughlin osteotomy of fifth metatarsal)

### Multivariate analysis of primary outcome

The logistic regression model demonstrated moderate evidence that radiological recurrence of HV was 45% less likely with the use of one screw for fixation after ReveL; irrespective of age, sex, preoperative HVA, additional surgical technique for HV, time period, and BMI (OR_adjusted_ = 0.55 [95% CI 0.30–0.98], *p* = 0.043) (Table 2). It also showed moderate evidence that radiological recurrence of HV was 162% more likely with the use of additional surgical technique for HV than without the use of additional surgical technique for HV (OR_adjusted_ = 2.62 [1.24–5.52], *p* = 0.011).

**Table 2:** Logistic regression model for several factors associated with radiological recurrence of hallux valgus deformity (HV) (hallux valgus angle (HVA) > 15°) and the number of screws used for fixation after reversed L-shaped osteotomy (ReveL) for HV (*n* = 799)

Main effect of variable	Stratum-specific effect of variable	Category	Adjusted OR (95% CI)^*^	*p* value^†^
One screw after ReveL for HV				
		No	1.00 (reference)	
		Yes	0.55 (0.30–0.98)	0.043
Additional surgical technique for HV				
		No	1.00 (reference)	
		Yes	2.62 (1.24–5.52)	0.011
Time period				
		2004–2007	1.00 (reference)	
		2008–2013	1.50 (0.91–2.47)	0.115

Abbreviations: *OR* odds ratio, *%* percent, *CI* confidence interval, *ReveL* reversed L-shaped osteotomy, *HV* hallux valgus deformity

^*^Adjusted for confounders and effect modifiers: age, sex, preoperative hallux valgus angle, number of screws, additional surgical technique for hallux valgus, time period, and body mass index. Note: The effect modifier Akin osteotomy was not included in the final model since it is already included in additional surgical techniques for hallux valgus

^†^Wald test

### Multivariate analysis of secondary outcomes

There was no evidence of an association of the secondary outcome variables (limited patient satisfaction, complication, revision surgery, and elective hardware removal) with the number of screws used for fixation after ReveL in the multivariate analysis (Table 3).

**Table 3:** Characteristics of cases treated with reversed L-shaped osteotomy (ReveL) for hallux valgus deformity using one or two screws and their association with secondary outcome variables (*n* = 799)

		One screw		
Variable	Category	No	Yes	*p* value^*^
Limited patient satisfaction^†^				
	No, *N* (%)	402 (83.4)	273 (86.2)	
	Yes, *N* (%)	80 (16.6)	44 (13.8)	
	OR_adjusted_ (95% CI)^‡^	1.00 (reference)	0.64 (0.37–1.08)	0.096
Complication^§^				
	No, *N* (%)	457 (94.8)	305 (96.2)	
	Yes, *N* (%)	25 (5.2)	12 (3.8)	
	OR_adjusted_ (95% CI)^‡^	1.00 (reference)	0.50 (0.20–1.24)	0.133
Revision surgery				
	No, *N* (%)	467 (96.9)	312 (98.4)	
	Yes, *N* (%)	15 (3.1)	5 (1.6)	
	OR_adjusted_ (95% CI)^‖^	1.00 (reference)	0.39 (0.07–2.16)	0.279
Elective hardware removal				
	No, *N* (%)	355 (73.7)	229 (72.2)	
	Yes, *N* (%)	127 (26.3)	88 (27.8)	
	OR_adjusted_ (95% CI)^‡^	1.00 (reference)	0.86 (0.56–1.33)	0.503

Abbreviations: *N* number, *%* percent, *OR* odds ratio, *CI* confidence interval

^*^Wald test

^†^Limited patient satisfaction was defined as dissatisfied or improved, while patient satisfaction was defined as satisfied or very satisfied

^‡^Adjusted for potential confounders and effect modifiers: age, sex, body mass index, additional surgical technique for hallux valgus, preoperative hallux valgus angle, and time period

^§^Defined as infection, osseous necrosis, non-union, complex regional pain syndrome, and revision

^‖^Adjusted for potential confounders and effect modifiers: age, sex, body mass index, additional surgical technique for hallux valgus, concomitant diseases, bilateral surgery, preoperative hallux valgus angle, preoperative distal metatarsal angle, and time period (*n* = 753 due to collinearity)

No evidence of associations of limited patient satisfaction with the number of screws used for fixation after ReveL (OR_adjusted_ = 0.64 [95% CI 0.37–1.08], *p* = 0.096), additional surgical technique for HV (OR_adjusted_ = 0.74 [95% CI 0.47–1.18], *p* = 0.202), and time period (OR_adjusted_ = 1.69 [95% CI 0.97–2.94], *p* = 0.062) were noted.

There was no evidence for associations of complications with the number of screws after ReveL (OR_adjusted_ = 0.50 [95% CI 0.20–1.24], *p* = 0.133), additional surgical technique for HV (OR_adjusted_ = 0.54 [95% CI 0.71–3.31], *p* = 0.272), and time period (OR_adjusted_ = 1.51 [95% CI 0.59–3.88], *p* = 0.391).

No evidence for associations of revision surgery with the number of screws after ReveL (OR_adjusted_ = 0.39 [95% CI 0.69–2.16], *p* = 0.279), additional surgical technique for HV (OR_adjusted_ = 1.23 [95% CI 0.42–3.62], *p* = 0.710), and time period (OR_adjusted_ = 1.18 [95% CI 0.26–5.47], *p* = 0.830) were observed.

**Fig. 4:**
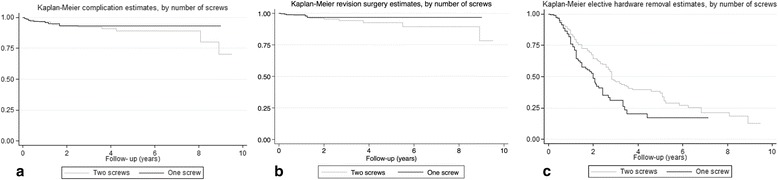
Kaplan-Meier curves. **a** Kaplan-Meier curve for complication after reversed L-shaped osteotomy (ReveL) for hallux valgus deformity (HV) (*p* = 0.902) (*n* = 810). **b** Kaplan-Meier curve for revision surgery after reversed L-shaped osteotomy (ReveL) for HV (*p* = 0.453) (*n* = 810). **c** Kaplan-Meier curve for elective hardware removal after reversed L-shaped osteotomy (ReveL) for HV (*p* = 0.001) (*n* = 810)

There was no evidence of associations of elective hardware removal with the number of screws (OR_adjusted_ = 0.86 [95% CI 0.56–1.33], *p* = 0.503), additional surgical techniques for HV (OR_adjusted_ = 1.14 [95% CI 0.78–1.66], *p* = 0.492), and time period (OR_adjusted_ = 1.20 [95% CI 0.76–1.89], *p* = 0.492). The cumulative probability of complication and revision surgery did not differ between cases with one screw and those with two screws after ReveL (*p* = 0.902 and *p* = 0.453, respectively) (Fig. 4a, b). The cumulative probability of no elective hardware removal was lower in cases with one screw after ReveL than in those with two screws after ReveL (*p* = 0.001) (Fig. 4c). The median time to hardware removal was shorter in cases with one screw after ReveL (12.0 [IQR 8.0–19.0] months) than those with two screws after ReveL (14.5 [9.0–32.0] months).

## Discussion

In this Swiss single-center cohort study, after adjustment for confounders, there were independent associations of the use of one screw for fixation after ReveL for HV and less radiological recurrence of HV as well as the use of additional surgical techniques for HV and increased radiological recurrence of HV. There were no associations of the number of used screws with limited patient satisfaction, complication, revision surgery, and elective hardware removal.

The literature about ReveL is very limited, and the level of evidence of the relevant studies is low [20, 29–33]. A previous small study showed that time to healing was faster with two screws compared to one screw after chevron osteotomy, but another small study did not find any differences [14, 15]. Our results add to this literature in that we found an independent association between the number of screws and radiological recurrence of HV, but not with the clinical outcome after ReveL. Although additional surgical techniques were described to have higher correction of HV, our results question their use if not absolutely necessary for substantially better correction of HV since they were associated with higher radiological recurrence of HV [16].

The evidence of an association between the number of screws with radiological recurrence of HV may be present since deforming forces that act on the first ray may be lower than those needed for failure of fixation. In other words, one screw may be strong enough to hold the osteotomy site in place. Similarly, no evidence of an association between the number of screws with clinical outcome may have been found since one screw may not be associated with less soft tissue irritation, swelling, and foreign body sensation. Although validation of the following is needed in further studies, one screw may potentially be considered for ReveL for HV due to its lower cost (23.60 Swiss Francs for one screw (2.4 mm cortex screw; Synthes, PA, USA)).

It is plausible that additional surgical techniques for HV are associated with increased radiological recurrence of HV because risk of failure inevitably increases with the number of osteotomies and fixations due to a more fragile overall construct. This fact could be used by surgeons when intraoperative ambiguity arises whether or not an additional surgical technique for HV should be used. This is clinically relevant because surgeons often contemplate whether correction of HV is sufficient or if an additional technique for HV could improve correction.

The fact that one screw was removed faster than two screws could potentially point toward faster bone healing entailing faster elective hardware removal since increasing tissue irritation by one screw is unlikely.

Previous studies about long plantar arm osteotomies consisted of much smaller sample sizes without adjustment for confounders. We provide new findings about potential associations between the number of screws and additional surgical techniques on the radiological and clinical outcomes. This information can be used by patients and orthopedic surgeons during pre-, intra-, and postoperative planning. Hospitals and insurance companies may also be interested in knowing that costs and surgical time could potentially be reduced.

Future studies could perform a superiority randomised controlled trial in order to compare the radiological recurrence and clinical outcome of HV after ReveL using one vs two screws. If possible, only one surgeon should use the same surgical techniques without additional surgical techniques and the same number of screws, preferably one screw, in all patients. A factorial design could be added to confirm our borderline evidence that additional surgical techniques for HV are associated with increased radiological recurrence of HV.

There are several limitations to this study. First, random assignment of cases limiting selection bias can be assumed by only including cases, where the surgical report did not mention a specific reason for the chosen number of screws. However, the risk of recall bias of surgeons (i.e., failure to mention a specific reason for number of screws) remains, although it is likely to be small since surgical reports are immediately written after the surgery. If surgeons forgot to mention that there was a specific risk for two screws, differential misclassification may have led to underestimation of the strength of an association between radiological recurrence of HV and the number of screws.

Second, blinding of surgeons to the study hypothesis limiting performance bias, differential treatment response, and misclassification may be assumed since surgeons did not know that this study would be conducted later on. Patients were usually told preoperatively that osteotomies would be fixed with implant material, such as one or two screws. However, a certain risk for information bias remains due to the retrospective nature of this study.

Third, due to the long study period and nature of a teaching hospital, numerous different surgeons performed surgeries, and usually, ≥ 2 surgeons were present at each surgery, rendering stratification difficult due to too many strata with small numbers. This is a potential confounder that cannot directly be controlled. However, every surgical procedure is supervised by a responsible senior surgeon, who follows the principles of the team leader, which ensures high and similar quality of each surgery. Choosing the number of screws is also usually in accordance with the personal preference of the senior surgeon. Selection bias and attenuation of effects may have arisen if more difficult cases were surgically treated by more experienced surgeons with a personal preference for one screw or vice versa. To adjust for this potential issue, two time periods were chosen and controlled for. Since the time period was chosen according to the time where different staff of the foot and ankle team was working, different philosophies of fixation techniques were indirectly adjusted for. Lastly, cases differed in their follow-up time due to the retrospective nature of this study. Although surveillance bias may have been present, the follow-up of patients is usually very good, and therefore, the last follow-up can be assumed to equal the final result.

## Conclusions

In ReveL for HV, a single screw (instead of two screws) may be sufficient enough for a similar of even better outcome, which may also reduce costs. Additional surgical procedures for HV may be refrained from if possible. Due to limitations of a retrospective study, the results may need validation with clinical trials.

## Abbreviations

Δ: Difference
CI: Confidence interval
HV: Hallux valgus deformity
HVA: Hallux valgus angle
IQR: Interquartile range
OR: Odds ratio
ReveL: Reversed L-shaped osteotomy
vs: Versus
